# Long-Term Brain Structure and Cognition Following Bariatric Surgery

**DOI:** 10.1001/jamanetworkopen.2023.55380

**Published:** 2024-02-09

**Authors:** Emma Custers, Debby Vreeken, Robert Kleemann, Roy P. C. Kessels, Marco Duering, Jonna Brouwer, Theo J. Aufenacker, Bart P. L. Witteman, Jessica Snabel, Eveline Gart, Henk J. M. M. Mutsaerts, Maximilian Wiesmann, Eric J. Hazebroek, Amanda J. Kiliaan

**Affiliations:** 1 Department of Medical Imaging, Anatomy, and Radboud Alzheimer Center, Radboud University Medical Center, Donders Institute for Brain, Cognition, and Behavior, Nijmegen, the Netherlands; 2 Department of Bariatric Surgery, Vitalys, part of Rijnstate hospital, Arnhem, the Netherlands; 3 Department of Metabolic Health Research, Netherlands Organisation for Applied Scientific Research, Leiden, the Netherlands; 4 Donders Institute for Brain, Cognition and Behaviour, Radboud University, Nijmegen, the Netherlands; 5 Department of Medical Psychology and Radboudumc Alzheimer Center, Radboud university medical center, Nijmegen, the Netherlands; 6 Vincent van Gogh Institute for Psychiatry, Venray, the Netherlands; 7 Medical Image Analysis Center and Department of Biomedical Engineering, University of Basel, Basel, Switzerland; 8 Institute for Stroke and Dementia Research, Ludwig Maximilian University Hospital, Ludwig Maximilian University of Munich, Munich, Germany; 9 Department of Radiology and Nuclear Medicine, Amsterdam UMC, location VUmc, Amsterdam Neuroscience, Amsterdam, the Netherlands; 10 Division of Human Nutrition and Health, Wageningen University, Wageningen, the Netherlands

## Abstract

**Importance:**

Weight loss induced by bariatric surgery (BS) is associated with improved cognition and changed brain structure; however, previous studies on the association have used small cohorts and short follow-up periods, making it difficult to determine long-term neurological outcomes associated with BS.

**Objective:**

To investigate long-term associations of weight loss after BS with cognition and brain structure and perfusion.

**Design, Setting, and Participants:**

This cohort study included participants from the Bariatric Surgery Rijnstate and Radboudumc Neuroimaging and Cognition in Obesity study. Data from participants with severe obesity (body mass index [BMI; calculated as weight in kilograms divided by height in meters squared] >40, or BMI >35 with comorbidities) eligible for Roux-en-Y gastric bypass and aged 35 to 55 years were enrolled from a hospital specialized in BS (Rijnstate Hospital, Arnhem, the Netherlands). Participants were recruited between September 2018 and December 2020 with follow-up till March 2023. Data were collected before BS and at 6 and 24 months after BS. Data were analyzed from March to November 2023.

**Exposure:**

Roux-en-Y gastric bypass.

**Main Outcomes and Measures:**

Primary outcomes included body weight, BMI, waist circumference, blood pressure, medication use, cognitive performance (20% change index of compound *z*-score), brain volumes, cortical thickness, cerebral blood flow (CBF), and spatial coefficient of variation (sCOV). Secondary outcomes include cytokines, adipokines, depressive symptoms (assessed using the Beck Depression Inventory), and physical activity (assessed using the Baecke Questionnaire).

**Results:**

A total of 133 participants (mean [SD] age, 46.8 [5.7] years; 112 [84.2%] female) were included. Global cognition was at least 20% higher in 52 participants (42.9%) at 24 months after BS. Compared with baseline, at 24 months, inflammatory markers were lower (mean [SD] high-sensitivity C-reactive protein: 4.77 [5.80] μg/mL vs 0.80 [1.09] μg/mL; *P* < .001), fewer patients used antihypertensives (48 patients [36.1%] vs 22 patients [16.7%]), and patients had lower depressive symptoms (median [IQR] BDI score: 9.0 [5.0-13.0] vs 3.0 [1.0-6.0]; *P* < .001) and greater physical activity (mean [SD] Baecke score: 7.64 [1.29] vs 8.19 [1.35]; *P* < .001). After BS, brain structure and perfusion were lower in most brain regions, while hippocampal and white matter volume remained stable. CBF and sCOV did not change in nucleus accumbens and parietal cortex. The temporal cortex showed a greater thickness (mean [SD] thickness: 2.724 [0.101] mm vs 2.761 [0.007] mm; *P* = .007) and lower sCOV (median [IQR] sCOV: 4.41% [3.83%-5.18%] vs 3.97% [3.71%-4.59%]; *P* = .02) after BS.

**Conclusions and Relevance:**

These findings suggest that BS was associated with health benefits 2 years after surgery. BS was associated with improved cognition and general health and changed blood vessel efficiency and cortical thickness of the temporal cortex. These results may improve treatment options for patients with obesity and dementia.

## Key Points

**Question:**

Does bariatric surgery–induced weight loss have long-term associations with brain structure and function?

**Findings:**

In this cohort study including 133 adults with severe obesity who underwent bariatric surgery, cognitive function, inflammatory biomarkers, comorbidities, physical activity, and depressive symptoms were still improved 2 years after bariatric surgery. On neuroimaging, the temporal lobe showed changes in structure and function.

**Meaning:**

These findings suggest that bariatric surgery was associated with long-term health benefits, including improvements in comorbidities, inflammation, and cognition; moreover, higher cortical thickness and lower spatial coefficient of variation were found in the temporal lobe 2 years after surgery.

## Teaser

This cohort study investigates long-term associations of weight loss after bariatric surgery with cognition and brain structure and perfusion.

## Introduction

Obesity is a major health problem and is associated with comorbidities and sequelae, such as type 2 diabetes and hypertension.^1^ These diseases affect the brain, but obesity itself is also associated with cognitive dysfunction and structural brain changes.^2^ Moreover, obesity is associated with 60% to 90% increased risk of developing dementia compared with lean individuals (body mass index [BMI; calculated as weight in kilograms divided by height in meters squared] <25).^3^

Obesity is inversely associated with gray matter (GM) volume^3,4,5,6^ and white matter (WM) integrity^7^ and positively associated with WM hyperintensities (WMH).^8^ These brain changes might be induced by reduced cerebral blood flow (CBF), which often coincides with obesity.^9^ Cognitive functions, particularly domains of executive function, attention,^10,11,12^ and episodic and working memory,^13,14,15^ are associated with obesity, corresponding to changes in hippocampus and prefrontal regions.^16,17^

To reduce potential consequences of obesity on the brain, long-term weight loss is important. Bariatric surgery (BS) leads to rapid and sustainable weight loss and improves comorbidities.^18^ Moreover, BS-induced weight loss has been reported to be associated with improved brain function and structure.^19,20,21,22^ However, results are contradictory, underlying mechanisms remain largely unknown, and it is uncertain whether outcomes are long-lasting. Imbalance of adipokines and proinflammatory cytokines may be involved, as they impair CBF and therewith cause neurodegeneration,^23^ which may be reversible after BS.^21^

Our study aims to strengthen the field, using state-of-the-art magnetic resonance imaging (MRI), a larger cohort with extended follow-up, and correction for multiple testing. This approach enhances our understanding of the disease, contributing to development of treatment strategies for obesity and dementia.

## Methods

This cohort study was approved by the Committee on Research Involving Human Subjects for Arnhem region, Nijmegen and the institutional ethics committee of the Rijnstate hospital. This study was performed according to the Declaration of Helsinki “Ethical Principles of Medical Research Involving Human Subjects” and in agreement with the International Council for Harmonisation of Technical Requirements for Pharmaceuticals for Human Use Guideline for Good Clinical Practice. All participants signed written informed consent. The study was prospectively registered in the Netherlands Trial Registry.^24^ We followed the Strengthening the Reporting of Observational Studies in Epidemiology (STROBE) reporting guideline.

### Study Sample

Data were obtained from the Bariatric Surgery Rijnstate and Radboudumc Neuroimaging and Cognition in Obesity (BARICO) study. Participants aged between 35 and 55 years who were eligible for Roux-en-Y gastric bypass based on Fried guidelines^25^ were recruited at Rijnstate Hospital (Arnhem, the Netherlands) between September 2018 and December 2020. Neurological or severe psychiatric illnesses, pregnancy, and treatment with antibiotics, probiotics, or prebiotics were exclusion criteria. Extra MRI exclusion criteria were epilepsy, claustrophobia, pacemakers, defibrillators, nerve stimulators, infraorbital or intraocular metallic fragments, intracranial clips, cochlear implants, ferromagnetic implants, circumference above MRI space capacity, left handedness, and color blindness.

Cognition was assessed before BS (baseline) and at 6 and 24 months after BS using neuropsychological tests. MRI scans were obtained at baseline and 24 months after BS. At all time points, blood samples and anthropometric data were collected. Only participants who completed measurements at all time points were included (eFigure 1 in Supplement 1).

### Medical Examination

Anthropometric measurements included body weight, waist circumference (WC), BMI, and percentage total body weight loss (TBWL). Percentage of TBWL was defined as:
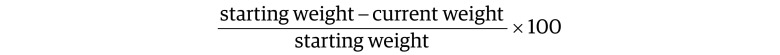
Blood pressure was measured in sitting position. Fasting blood samples were collected at baseline and 6 and 24 months after BS. Adipokines and cytokines were analyzed as described in the eMethods in Supplement 1.

### Cognition

Cognition was assessed using neuropsychological tests, described in detail elsewhere.^26^ To assess overall cognitive performance, we used the Montreal Cognitive Assessment (MOCA). We used the Digit Span test (Wechsler Adult Intelligence Scale–Fourth Edition) to measure working memory.^27^ Episodic memory was assessed with the immediate and delayed Story Recall subtest from the Rivermead Behavioral Memory Test.^28^ The Flexibility subtest from the computerized Test of Attentional Performance (version 2.3.1) was used to measure ability to shift attention.^29^ Verbal fluency was assessed using the Controlled Oral Word Association Test (COWAT).^30^ Parallel versions were used when appropriate to overcome material specific practice effects. Total scores of every subtest were converted and we calculated the mean into a compound *Z*-score of global cognitive performance, which ranged from −1.51 to 2.02, with higher score indicating higher cognitive performance. Education level was assessed by the Verhage score (with 1 indicating the lowest level, ie, less than primary school; and 7, the highest level of education, ie, university)^31^ based on the Dutch educational system, comparable with the International Standard Classification of Education.^32^ A score of 4 or less indicates low education level; 5, middle educational level; and 6 or 7, high educational level.

To examine the association of BS with cognition, while excluding practice effects, we calculated the 20% change index^33^ 24 months after BS. This index assumes that participants show clinically meaningful and significant cognitive improvement if their postoperative test score is 20% higher than the preoperative test score. To calculate this index, we used:
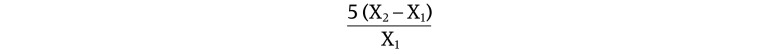
Where *X_2_* is participants’ postoperative score and *X_1_* the preoperative score. Calculations were performed for each domain and the composite *Z*-score. An index of 1.00 or greater indicated a significant improvement.^33^

### MRI Acquisition, Outcomes, and Image Processing

Participants were scanned in a 3T Skyra scanner (Siemens Healthineers) using a 32-channel head coil. The sequences used are listed the eMethods in Supplement 1.

#### Brain Volume and Cortical Thickness

Image reconstruction and segmentation were performed with default settings of the Freesurfer Imaging Analysis Suite version 6.0.0.^34^ Global measures included total cerebral GM and WM volumes (normalized by intracranial volume) and overall mean cortical thickness. Subcortical volumes (hippocampus, amygdala, caudate nucleus, putamen, and nucleus accumbens) and volume and thickness metrics for specific region of interests (ROIs) from the merged Desikan-Killiany atlas^35^ (frontal, occipital, parietal and temporal cortex, cingulate gyrus, and insula) were measured. As an additional analysis, we calculated cortical thinning 2 years after BS (eTable 1 in Supplement 1).

#### WMH and Integrity

A fully automated, deep learning algorithm used 3-dimensional (3D) fluid-attenuated inversion recovery (FLAIR) and 3D T1-weighted images to segment WMH using multidimensional gated recurrent units.^36^ Output WMH segmentation masks were used to determine WMH volume. Global WM mean diffusivity was assessed using diffusion MRI data processed as previously outlined.^37^ We calculated mean skeletonized mean diffusivity (MSMD) via an accessible method.^38^

#### Arterial Spin Labeling

Postprocessing of arterial spin labeling images was performed with toolbox ExploreASL,^39^ version 1.5.1, including SPM12, version 7219 (Statistical Parametric Mapping, Wellcome Trust Centre for Neuroimaging), CAT12 version r1615, and LST version 2.0.15, all operated in MATLAB version 2020a (MathWorks). Complete processing steps are described elsewhere.^39^

CBF and spatial coefficient of variation (sCOV) within overall GM and different ROIs were calculated. sCOV was determined by dividing the SD of CBF by mean CBF.^40^ ROIs were prespecified combining the Harvard-Oxford^35^ and Montreal Neurological Institute^41^ structural atlases: caudate nucleus, putamen, nucleus accumbens, insula, and frontal, occipital, parietal and temporal cortex. Due to field of view, we were not able to include more ventrally located regions, such as hippocampus and amygdala. CBF and sCOV were calculated with partial volume correction for overall GM and each ROI.^39^

### Questionnaires for Depressive Symptoms and Physical Activity

At baseline and 6 and 24 months after BS, participants filled out standardized online questionnaires. Depressive symptoms were assessed via the Beck Depression Inventory (BDI),^42^ which determines depressive symptoms over the past 2 weeks (range, 0-63; higher score indicates greater depressive symptoms). Physical activity was assessed with the Baecke Questionnaire,^43^ which incorporates time spent on different activities (range, 3-15; higher score indicates greater physical activity). All participants filled in the questionnaires, but not all questionnaires were complete, resulting in some missing data (eTable 2 in Supplement 1).

### Statistical Analysis

Explorative statistical analyses were performed using SPSS Statistics version 27 (IBM). Continuous variables were checked for normality. If normality was not met, natural log transformations were performed for repeated measures analyses of variance (ANOVA). To test changes in primary and secondary outcomes over time, repeated measures ANOVA with Bonferroni correction (to correct for multiple comparisons), Cochran test, Friedman test, Wilcoxon signed ranks test or χ^2^ tests were used for continuous and categorical data. We controlled for age, sex, education, and preoperative BMI in the repeated measures ANOVA. Additionally, we controlled for hematocrit and head motion for CBF and sCOV and head motion for MSMD. Missing variables are presented in eTable 2 in Supplement 1. Included covariates are listed in eTable 3 in Supplement 1. *P* values were 2-sided, and *P* < .05 was considered statistically significant. Data were analyzed from March to November 2023.

## Results

### Descriptive Statistics

A total of 133 participants (mean [SD] age, 46.8 [5.7] years; 112 [84.2%] female) were included. Participants characteristics are listed in Table 1. Overall, mean body weight, BMI, WC, and blood pressure were significantly lower 6 and 24 months after BS (Table 1). From 6 to 24 months, percentage TBWL was significantly higher. Compared with baseline, medication use for comorbidities was significantly lower 24 months after surgery (eg, antihypertensive use, 48 patients [36.1%] vs 22 patients [16.7%]).

**Table 1.** Characteristics of Participants

Characteristic	Participants, No. (%)											
	All participants (N = 133)				Male (n = 21)				Female (n = 112)			
	Baseline	6 mo	24 mo	*P* value	Baseline	6 mo	24 mo	*P* value	Baseline	6 mo	24 mo	*P* value
Age, mean (SD), y	46.8 (5.7)	NA	NA	NA	48.9 (5.0)	NA	NA	NA	46.3 (5.8)	NA	NA	NA
Body height, mean (SD), m	1.71 (0.07)	1.71 (0.07)	1.71 (0.07)	NA	1.81 (0.05)	1.81 (0.05)	1.81 (0.05)	NA	1.70 (0.06)	1.70 (0.06)	1.70 (0.06)	NA
Body weight, mean (SD), kg	122.46 (15.75)	89.20 (12.61)	80.45 (13.27)	<.001	140.08 (13.45)	100.79 (11.62)	95.77 (11.6)	<.001	119.17 (13.89)	87.03 (11.60)	77.58 (11.76)	<.001
BMI, mean (SD)	41.87 (4.11)	30.46 (3.71)	27.44 (3.77)	<.001	42.52 (3.69)	30.51 (3.18)	29.01 (2.71)	<.001	41.75 (4.19)	30.45 (3.81)	27.15 (3.88)	<.001
WC, mean (SD), cm^a^	125.14 (11.96)	100.57 (10.91)	93.67 (10.46)	<.001	137.47 (6.77)	107.81 (9.87)	102.62 (8.77)	<.001	122.73 (11.20)	99.45 (10.98)	94.79 (10.58)	<.001
TBWL, mean (SD), %	NA	27.12 (5.03)	34.26 (7.03)	<.001	NA	28.09 (3.74)	31.49 (5.03)	.002	NA	26.94 (5.23)	34.78 (7.25)	<.001
Level of education^b^												
Low	13 (9.8)	NA	NA	NA	3 (14.3)	NA	NA	NA	10 (8.9)	NA	NA	NA
Middle	70 (52.6)	NA	NA	NA	11 (52.4)	NA	NA	NA	60 (53.6)	NA	NA	NA
High	49 (36.8)	NA	NA	NA	7 (33.3)	NA	NA	NA	42 (37.5)	NA	NA	NA
Use of medication^c^												
Oral antidiabetics	12 (9.0)	8 (6.0)	3 (2.3)	.009	3 (14.3)	2 (9.5)	0 (0)	.10	9 (8.0)	6 (5.4)	3 (2.7)	.07
Insulin therapy	7 (5.3)	3 (2.3)	3 (2.3)	.02	0 (0)	0 (0)	0 (0)	NA	7 (6.3)	3 (2.7)	3 (2.7)	.02
BP-lowering agents	48 (36.1)	31 (23.3)	22 (16.7)	<.001	12 (57.1)	9 (42.9)	8 (40.0)	.10	36 (32.1)	22 (19.6)	14 (12.5)	<.001
Lipid-lowering agents	19 (14.3)	12 (9.0)	12 (9.0)	.004	6 (28.6)	5 (23.8)	5 (23.8)	.37	13 (11.6)	7 (6.3)	7 (6.3)	.01
Antidepressants	14 (10.5)	10 (7.5)	12 (9.0)	.18	1 (4.8)	0 (0)	0 (0)	.37	13 (11.6)	10 (8.9)	12 (10.7)	.31
BP, median (SD), mm Hg^d^												
Systolic	137.33 (15.99)	126.81 (17.62)	129.66 (18.89)	<.001	144.86 (18.88)	132.88 (22.19)	141.00 (24.40)	.25	135.89 (14.65)	125,28 (16.33)	127.75 (17.50)	<.001
Diastolic	85.25 (8.55)	80.78 (11.34)	80.49 (11.93)	<.001	90.19 (13.47)	85.41 (16.0)	85.90 (13.60)	.54	84.68 (7.10)	79.59 (9.94)	79.69 (11.45)	<.001
MOCA score, median (IQR)^e^	27.0 (26.0-29.0)	27.0 (25.0-28.0)	27.0 (25.0-28.0)	<.001	25.8 (24.0-27.0)	26.4 (25.0-28.0)	24.9 (24.0-26.5)	.32	28.0 (26.0-29.0)	27.0 (25.0-28.0)	27.0 (25.0-28.0)	<.001

Abbreviations: BMI, body mass index; BP, blood pressure; MOCA, Montreal Cognitive Assessment; NA, not applicable; TBWL, total body weight loss; WC, waist circumference.

^a^ Complete data on all time points were available for 94 participants (15 male participants; 79 female participants).

^b^ A Verhage score of 4 or less is defined as a low level of education; 5, middle level; and 6 or 7, high level.^31^

^c^ Complete data on all time points were available for 132 participants (20 male participants; 112 female participants). Cochran Test was conducted to assess changes over time.

^d^ Complete data on both time points were available for 97 participants (17 male participants; 80 female participants). Corrected for antihypertensives.

^e^ Friedman Test was conducted to assess changes over time.

### Changes in Cognition, Depressive Symptoms, and Physical Activity

Several cognitive domains significantly improved at 6 and 24 months after BS (Table 2; eFigure 2 in Supplement 1). At baseline our cohort had a median (IQR) MOCA score of 27 (26.0-29.0). Nonetheless, based on the 20% change index, 15 participants (11.3%) showed improvements in working memory, 42 participants (31.6%) showed improvements in episodic memory, 32 participants (24.1%) showed improvements in in verbal fluency, 51 participants (40.2%) showed improvements in ability to shift attention, and 52 participants (42.9%) showed improvements in global cognition. According to the BDI score at baseline, 71 participants (54.6%) experienced minimal depressive symptoms, 55 participants (42.3%) experienced mild depressive symptoms, and 4 participants (3.1%) experienced moderate depressive symptoms. At 24 months after BS, 12 participants (9.4%) had mild depressive symptoms and 2 participants (1.6%) had moderate depressive symptoms. Additionally, the Baecke score was significantly higher 6 months after surgery and remained stable up to 24 months (mean [SD] Baecke score: baseline, 7.64 [1.29]; 6 mo, 8.36 [1.23]; 24 mo, 8.19 [1.35]; *P* < .001).

**Table 2.** Changes in Cognition, Depression Symptoms, and Physical Activity Among Patients Who Underwent Bariatric Surgery

Measure	Baseline	6 mo	24 mo	*P* value	Individuals with ≥20% change, No. (%)
Cognition, mean (SD)					
Digit span (sum of forward, backward, and sorting)	25.95 (4.92)	26.47 (4.52)	26.80 (4.91)	.02	15 (11.3)
Story Recall (sum of immediate and delayed recall)	16.98 (6.51)	18.56 (6.60)	17.16 (6.01)	.003	42 (31.6)
COWAT	37.70 (10.74)	40.94 (11.68)	40.69 (11.69)	<.001	32 (24.1)
TAP flexibility index score^a^	−3.04 (8.53)	0.94 (8.43)	2.15 (7.83)	<.001	51 (40.2)
Compound *Z*-score^a^	0.02 (0.68)	0.28 (0.66	0.29 (0.68)	<.001	52 (42.9)
BDI^b^					
Median (IQR)	9.0 (8.0)	5.0 (4.0)	3.0 (5.0)	<.001	NA
Group, No. (%)					
Minimal	71 (54.6)	110 (85.9)	114 (89.1)	NA	NA
Mild	55 (42.3)	18 (14.1)	12 (9.4)	NA	NA
Moderate	4 (3.1)	0	2 (1.6)	NA	NA
Severe	0	0	0	NA	NA
Baecke, mean (SD)^c^	7.64 (1.29)	8.36 (1.23)	8.19 (1.35)	<.001	NA

Abbreviations: BDI, Beck Depression Inventory; COWAT, Controlled Oral Word Association Test; NA, not applicable; TAP, Test of Attentional Performance.

^a^ Complete data on all time points were available for 117 participants.

^b^ Friedman Test was conducted to assess changes over time. Complete data on all time points were available for 124 participants.

^c^ Complete data on all time points were available for 91 participants.

### Changes in Brain Parameters

Brain changes were observed after BS (Table 3). GM volume, GM cortical thickness, and GM CBF were significantly lower 2 years after BS. Several other ROIs, including amygdala, caudate nucleus, putamen, insula, cingulate gyrus, and occipital, parietal, and temporal cortex exhibited significantly lower volumes after BS. No volumetric changes were observed in hippocampus, nucleus accumbens, frontal cortex, or WM. Cortical thickness of all ROIs was significantly lower after BS, except thickness of the temporal cortex, which was significantly larger (mean [SD] thickness: 2.724 [0.101] mm vs 2.761 [0.007] mm; *P* = .007). Moreover, after BS, CBF was lower in several cortical and subcortical regions, including caudate nucleus, putamen, insula, and frontal and occipital cortex. CBF in temporal cortex, parietal cortex, and nucleus accumbens did not change after BS. Regarding sCOV, the caudate nucleus showed a higher sCOV, while temporal cortex showed lower sCOV after BS (median [IQR] sCOV: 4.41% [3.83%-5.18%] vs 3.97% [3.71%-4.59%]; *P* = .02). sCOV of all other ROIs remained stable over time. MSMD was significantly lower, whereas WMH volume did not change after BS.

**Table 3.** Change in Brain Parameters Among Patients Who Underwent Bariatric Surgery (n = 63)

Measure	Mean (SD)		*P* value
	Baseline	24 mo	
Brain volume, % ICV^a^			
Hippocampus	0.572 (0.042)	0.570 (0.041)	.31
Amygdala	0.233 (0.022)	0.229 (0.019	.002
Nucleus accumbens	0.067 (0.009)	0.064 (0.009)	.86
Caudate nucleus	0.481 (0.047)	0.473 (0.047)	<.001
Putamen	0.668 (0.062)	0.657 (0.060)	<.001
Cingulate gyrus	1.234 (0.094	1.184 (0.101)	<.001
Frontal cortex	10.783 (0.700)	10.294 (0.810)	.56
Insula	0.870 (0.660)	0.85 (0.059)	<.001
Occipital cortex	2.868 (0.335)	2.791 (0.284)	.002
Parietal cortex^b^	7.384 (0.613)	7.149 (0.690)	<.001
Temporal cortex	6.501 (0.511)	6.222 (0.565)	<.001
WM	31.889 (1.844)	32.034 (1.747)	.13
GM	41.337 (2.134)	40.707 (2.021)	<.001
Cortical thickness, mm^a^			
Cingulate gyrus	2.398 (0.084)	2.369 (0.075)	<.001
Frontal cortex	2.552 (0.010)	2.510 (0.091)	<.001
Insula	2.884 (0.106)	2.859 (0.101)	.01
Occipital cortex	1.935 (0.089)	1.902 (0.098)	.004
Parietal cortex^b^	2.392 (0.091)	2.361 (0.089)	<.001
Temporal cortex	2.724 (0.101)	2.761 (0.110)	.007
GM	2.450 (0.085)	2.413 (0.082)	<.001
CBF, mL/100 g/min^c^			
Nucleus accumbens^d^	37.008 (14.002)	34.539 (14.571)	.08
Caudate nucleus	36.044 (9.859)	31.836 (9.957)	.003
Putamen	38.560 (8.583)	34.349 (7.790)	.001
Frontal cortex	47.205 (8.788)	43.487 (8.870)	.001
Insula	47.065 (8.516)	42.672 (8.110)	<.001
Occipital cortex	51.865 (8.564)	48.252 (8.806)	.008
Parietal cortex^e^	51.402 (8.924)	49.643 (9.089)	.14
Temporal cortex	52.643 (8.850)	49.615 (10.270)	.07
GM	46.213 (7.686)	42.671 (7.803)	<.001
sCOV, median (IQR), %^c^			
Nucleus accumbens^d^	4.29 (3.31-6.16)	4.32 (3.32-6.67)	.95
Caudate nucleus	3.82 (3.13-4.55)	3.89 (3.18-5.13)	.03
Putamen	2.36 (2.12-2.30)	2.58 (2.21-3.08)	.35
Frontal cortex	3.63 (3.05-4.40)	3.62 (3.31-4.37)	.94
Insula	3.03 (2.74-3.42)	3.16 (2.78-3.68)	.65
Occipital cortex	5.78 (5.16-7.01)	5.45 (4.80-6.41)	.19
Parietal cortex	4.90 (4.42-5.86)	4.89 (4.34-5.63)	.59
Temporal cortex	4.41 (3.83-5.18)	3.97 (3.71-4.59)	.02
GM	3.92 (3.45-4.59)	4.00 (3.56-4.49)	.96
WM integrity			
MSMD, 10^−4^ mm^2^/s	3.387 (0.108)	3.446 (0.103)	<.001
WMH volume, median (IQR), mL	0.108 (0.025-0.258)	0.103 (0.033-0.256)	.12

Abbreviations: CBF, cerebral blood flow; GM, gray matter; ICV, intracranial volume; MSMD, mean skeletonized mean diffusivity; sCOV, spatial coefficient of variation; WM, white matter; WMH, white matter hyperintensity.

^a^ Complete data on both time points were available for 61 participants.

^b^ Complete data on both time points were available for 60 participants.

^c^ Complete data on both time points were available for 59 participants.

^d^ Complete data on both time points were available for 49 participants.

^e^ Complete data on both time points were available for 58 participants.

### Changes in Adipokines and Inflammatory Factors

**Figure 1.**
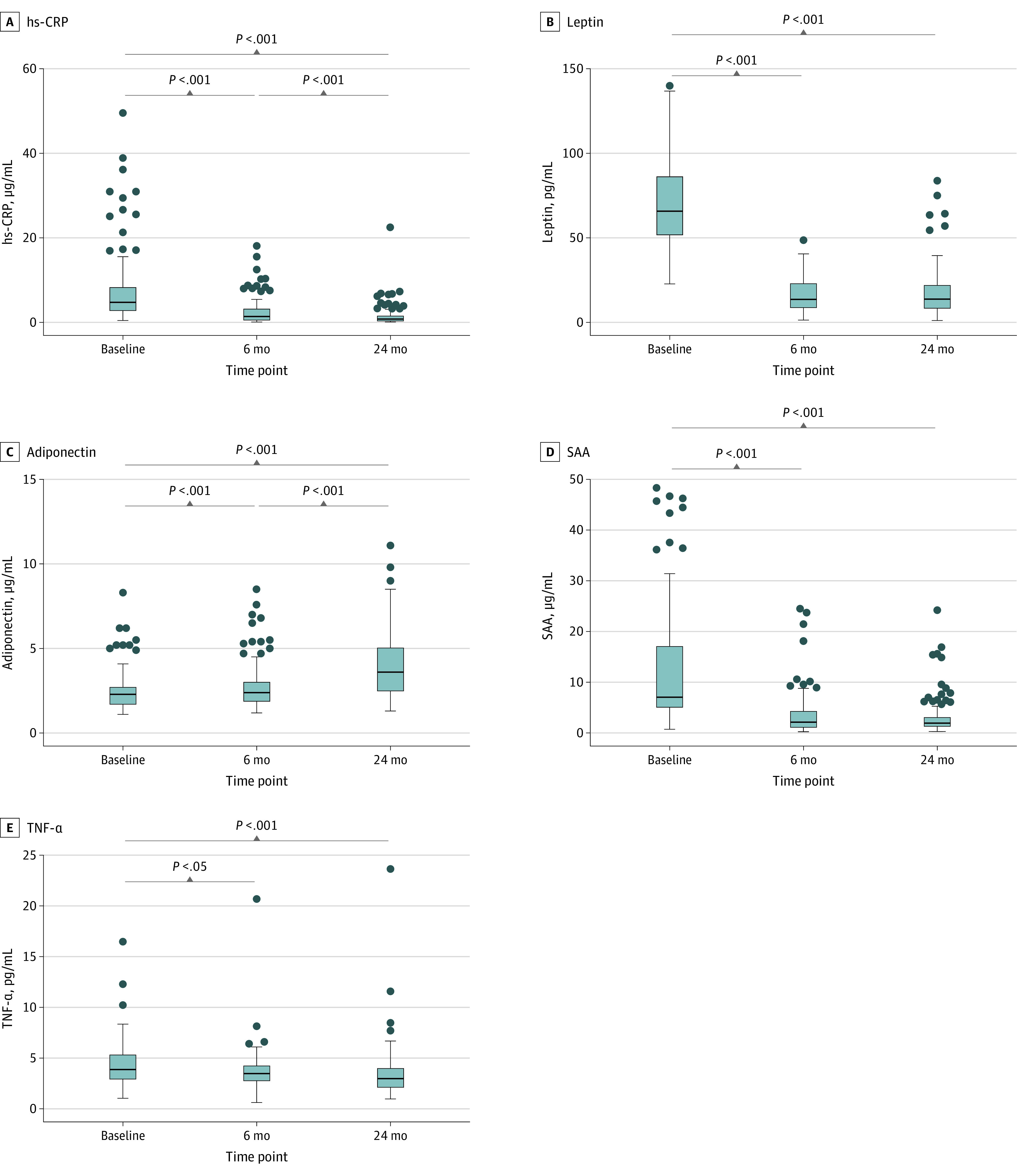
Plasma Concentrations of Adipokines and Cytokines Among Patients Who Underwent Bariatric Surgery Repeated measures analysis of variance were conducted to assess changes in circulating factors over time. Significant changes over time are indicated by *P* values. Complete data for all parameters on both time points were available for 110 participants. For illustrative purposes 3 extreme high SAA values (111.49 µg/mL, 401.25, and 50.70 µg/mL) at baseline are not shown. Individual values for every plasma marker are presented in eTable 4 in Supplement 1. hs-CRP indicates high sensitive C-reactive protein; SAA, serum amyloid A; TNF-α, tumor necrosis factor α. Dots indicate individual data points; bars, medians; bars, IQRs; and whiskers, ranges.

**Figure 2.**
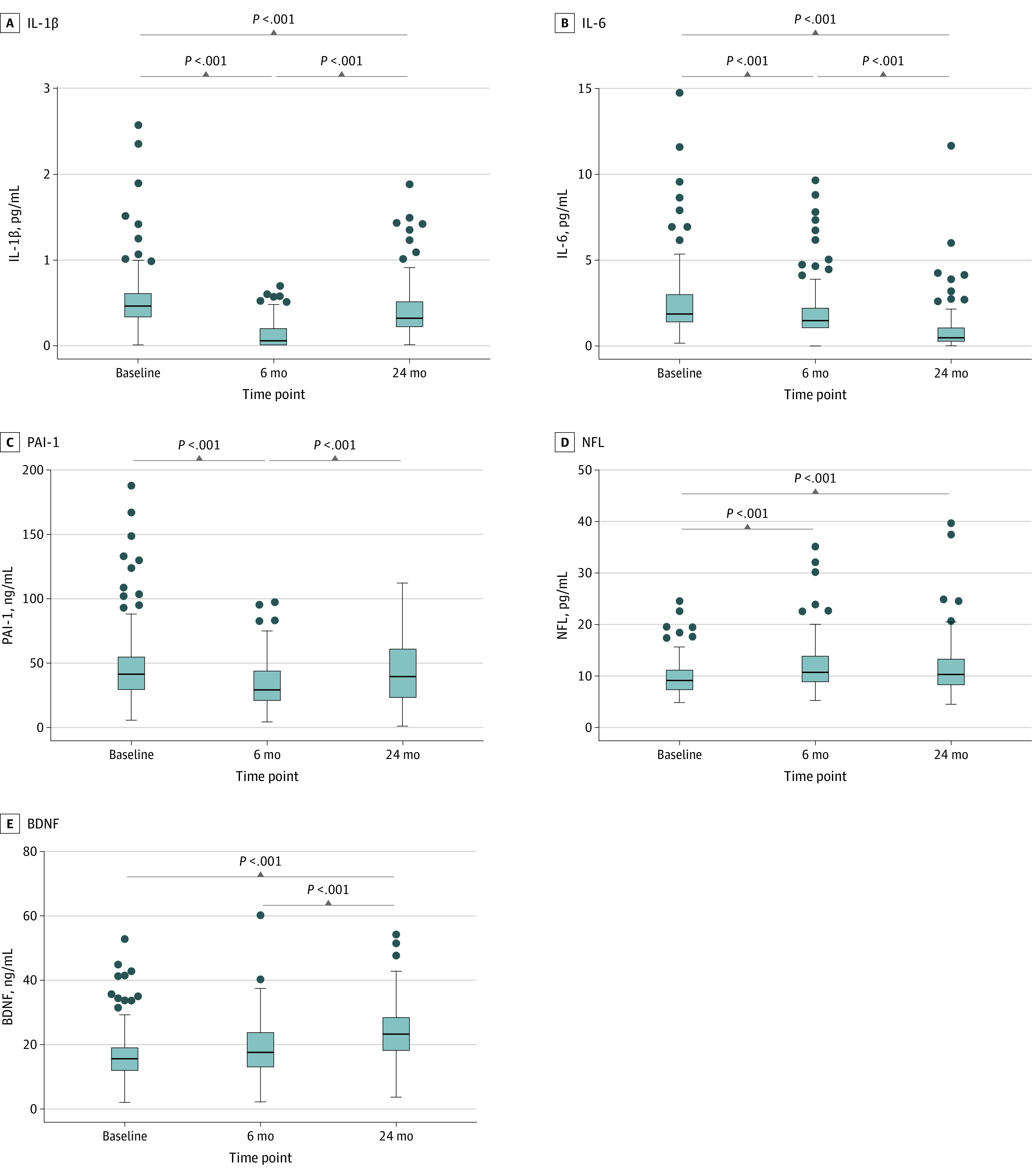
Plasma Concentrations of Cytokines and Brain-Associated Blood-Based Biomarkers Among Patients Who Underwent Bariatric Surgery Repeated measures analysis of variance were conducted to assess changes in circulating factors over time. Significant changes over time are indicated by *P* values. Complete data for all parameters on both time points were available for 110 participants, except for interleukin-1β (IL-1β), for which data from 111 participants were available. For illustrative purposes 3 extreme high IL-1β values (7.59 pg/mL, 12.75 pg/mL, and 3.94 pg/mL) at baseline are not shown. At the 2 year time point, 2 extreme high IL-1β values (5.12 and 3.26 pg/mL) was not included for illustrative purposes. Individual values for every plasma marker are presented in eTable 4 in Supplement 1. BDNF indicates brain derived neurotrophic factor; NFL = neurofilament light chain; PAI-1 plasminogen activator inhibitor-1. Dots indicate individual data points; bars, medians; bars, IQRs; and whiskers, ranges.

Circulating markers were analyzed before and after surgery (Figure 1 and Figure 2; eTable 4 in Supplement 1). After 6 months, high-sensitivity C-reactive protein (hs-CRP), leptin, serum amyloid A, tumor necrosis factor–α, interleukin-1β (IL-1β), IL-6, and plasminogen activator inhibitor-1 were significantly lower, whereas adiponectin and neurofilament light chain (NFL) were significantly higher compared with baseline. hs-CRP and IL-6 were still lower at 24 months (eg, mean [SD] hs-CRP: baseline, 4.77 [5.80] μg/mL vs 0.80 [1.09] μg/mL; *P* < .001), while leptin, serum amyloid A, and tumor necrosis factor–α did not change at 24 months compared with 6 months after BS. Surprisingly, plasminogen activator inhibitor-1 returned to baseline levels by 24 months after BS. IL-1β was higher 24 months after BS compared with the 6-month follow-up but remained significantly lower compared with baseline. At 24 months after BS, adiponectin was higher, while NFL remained stable compared with the 6-month follow-up. brain-derived neurotrophic factor (BDNF) was significantly higher at 24 months after BS.

## Discussion

This cohort study investigated the associations of BS-induced weight loss with obesity-related comorbidities, physical activity, mood, cognition, brain parameters, and circulating factors 2 years after surgery. We found that 42.9% of the participants improved at least 20% in global cognitive function. Additionally, BS was associated with less medication use and depressive symptoms and more physical activity. The stabilization (ie, no changes over time) of cerebral structures and functions was the most noteworthy finding. While volumes and perfusion were lower in many brain regions after surgery, some regions exhibited stability. Despite the lower CBF in several regions, volumes of hippocampus, nucleus accumbens, frontal cortex, WM, and WMH remained stable after surgery. Notably, the temporal cortex exhibited not only higher cortical thickness but also higher vascular efficiency after surgery, as indicated by a lower sCOV. These results highlight beneficial vascular responses occurring in conjunction with BS. Accordingly, nucleus accumbens and parietal cortex demonstrated stable CBF and cerebrovascular efficiency. After BS, general health also changed, including lower blood pressure, lower inflammatory markers, lower leptin, and higher adiponectin levels. Finally, higher brain-associated blood-based biomarkers for axonal damage (ie, NFL) and neurogenesis (ie, BDNF) were observed.

High scores on MOCA and other neuropsychological tests were obtained at baseline, suggesting that obesity did not impair cognitive performance in clinical sense. Yet, as previously described,^44,45,46^ cognition improved significantly after BS, with the largest improvements observed in attention and verbal fluency, components that can be impaired in obesity^47^ but may be reversible after BS.^48^ We observed significantly improved performance in all cognitive domains at 6 months after BS, and these improvements (except for episodic memory) lasted to the 24-month postsurgery follow-up. These findings suggest that cognitive improvements begin shorty after BS and are long lasting. Various factors may be involved, including remission of comorbidities, higher physical activity, lower depressive symptoms, and lower inflammatory factors after BS.^22^ Additionally, stabilization of volume, CBF, and sCOV in brain regions, together with larger cortical thickness and higher vascular efficiency in the temporal cortex, might be involved.

This study found associations of BS with brain parameters 2 years after surgery. BS was associated with lower cortical volumes and thickness in some ROIs. We assume that aging was involved. The relative mean change in global GM (−0.6%) in our study is comparable with aging studies.^49,50^ When applying this aging rate of 0.6% on cortical thickness, cortical thinning in GM was higher in our study. However, for temporal cortex and insula, less cortical thinning was observed after surgery. Furthermore, MRI studies focusing on regional distribution of aging-related GM volumetric reductions have reported large changes in frontal and temporal lobe.^51^ In our study, frontal lobe volumes did not change and temporal cortical thickness was higher after surgery, suggesting that BS might delay aging-related decline in some regions. Similarly, WM volumes are relatively stable over time in middle-aged individuals,^51^ suggesting that WM volumetric changes are not accelerated by obesity. Moreover, higher NFL and BDNF levels were observed 2 years after surgery. Serum NFL is a marker associated with neuroaxonal damage and reflects WM integrity.^52^ NFL levels were significantly higher at 24 months after BS, but still lower compared with individuals without neurological anomalies in the same age range.^53^ This suggests little axonal damage and that lower MSMD values were not yet reflected in circulating levels. BDNF is a neurotrophic factor involved in survival and plasticity of neurons.^54^ It is decreased in obesity^55^ and Alzheimer disease^56^ and is positively associated with WM volume.^57^ In this study, participants showed higher BDNF levels after BS, highlighting its potential role in cognition, mood, and protection of WM degeneration.

At baseline and 24 months after surgery, our cohort showed a lower CBF compared with participants with weight within reference range in a study by Chen et al.^58^ However, our arterial spin labeling results revealed promising outcomes in certain brain regions. CBF was significantly lower in GM, caudate nucleus, putamen, insula, and frontal and occipital cortex after surgery. Contrastingly, in nucleus accumbens, parietal, and temporal cortex, CBF remained stable after surgery, signifying a favorable outcome associated with BS. sCOV was higher in caudate nucleus, and lower in the temporal cortex 24 months after surgery. It is noteworthy that a higher sCOV indicates lower vascular efficiency of the blood vessels.^59^ The finding that the temporal cortex showed no change in CBF level but higher vascular efficiency might be due to lower inflammatory markers and lower blood pressure. Nonetheless, decline in CBF in most brain regions surpassed the aging-related decline observed in healthy participants with an age range between 22 and 82 years.^60,61^ Obesity is associated with vascular pathologies^7^ that affect vessel quality, thereby increasing sCOV. Probably, these vascular alterations and corresponding perfusion irregularities are not yet reversible at 24 months after BS, which could explain lower CBF levels after surgery. Moreover, we assume that lower CBF levels contribute to structural brain alterations in some ROIs, as CBF plays a crucial role in maintenance of GM and WM.^62^ Notably, previous aging studies investigated healthy individuals, whereas this study assessed brain changes in people with a history of obesity. Furthermore, these studies used different MRI acquisition and postprocessing methods, which could induce different results. Nevertheless, we suggest that aging brain outcomes, as observed in aging studies, are accelerated in individuals with a history of obesity, but might be stabilized or improved in certain ROIs following BS.

Remarkably, other studies have detected increased brain volumetry after BS,^19,20^ while we identified lower or stabilized volumes after surgery. We used high-quality 3T imaging, and cerebral spinal fluid partial volume outcomes were excluded, which could explain different results. Moreover, the smaller cohorts and shorter follow-ups of those studies could be influencing factors. Furthermore, voxel-based morphometry, as used by others,^19,20^ and Freesurfer may reveal differences in brain volume reduction, as they use distinct analysis approaches. Additionally, brain volume reduction differs per region and can be influenced by various additional factors.^63^

### Limitations

Our study has limitations. First, we did not include a control group, making it difficult to conclude whether outcomes were associated with aging or prolonged obesity. We therefore attempted to compare results with aging data from other studies, striving to discern age-related changes independently of the potential influence of prolonged obesity. Second, our study had an unequal sex distribution, with less than 20% of the sample being male. This is important to consider, as brain atrophy is greater in women than in men.^64^ However, the sex distribution of our sample represents the general BS population.^65^ Third, cortical surface and curvature (parameters obtainable by Freesurfer) were not included. These parameters could improve our understanding of change in cortical volume and thickness after BS. Strengths of the study include a large sample size, a long follow-up and use of standardized and parallel versions of cognitive tests and the 20% change index to control for practice effects. Additionally, we included measures on adipokines and cytokines and information on physical activity and mood to elucidate potential factors influencing for changes associated with BS.

## Conclusions

The results of this cohort study indicate that cognitive improvement was sustained in approximately 40% of participants at 24 months after BS, potentially due to lower inflammation and adipokine secretion, remission of comorbidities, higher physical activity, and better mood. These changes were reflected by stabilized or higher volumes, cortical thickness, and blood vessel efficiency in some ROIs. More specifically, the nucleus accumbens demonstrated stable CBF and sCOV, complementing the preserved volume, while the hippocampus and WM exhibited stability in volume. After surgery, a larger cortical thickness and lower sCOV were observed in the temporal cortex. Altogether, these results provide new information on longer-term outcomes associated with BS-induced weight loss in cognition and brain structure and perfusion, although exact underlying mechanisms remain unsolved. Future studies should include control groups and other mechanisms to clarify cognition and brain changes after BS. Such studies can contribute to development of strategies to reduce risk of obesity and neurodegenerative diseases.

## Notes

Supplement 1. **eFigure 1.** Flowchart of the Study **eMethods.** Analysis of Adipokines and Cytokines and MRI Acquisition **eTable 1.** Cortical Thinning Based on 0.6% Annual Normal Brain Aging Effects **eTable 2.** Missing Data Per Outcome Measure at Baseline and 6 Months After Bariatric Surgery **eTable 3.** Covariates Included in Repeated Measures ANOVA Analysis **eFigure 2.** Changes in Cognitive Outcomes, Physical Activity and Depression Symptoms Among Patients Who Underwent Bariatric Surgery (n=133) **eTable 4.** Change in Plasma Markers Among Patients Who Underwent Bariatric Surgery (n=133)
eReference.

Supplement 2.
Data Sharing Statement
