# Supplementary material for: Long-Term Brain Structure and Cognition Following Bariatric Surgery
Source: JAMA Netw Open. 2024 Feb 9;7(2):e2355380. doi: 10.1001/jamanetworkopen.2023.55380 (PMC10858407; doi:10.1001/jamanetworkopen.2023.55380)
Supplement: Supplement 1. — eFigure 1. Flowchart of the Study eMethods. Analysis of Adipokines and Cytokines and MRI Acquisition eTable 1. Cortical Thinning Based on 0.6% Annual Normal Brain Aging Effects eTable 2. Missing Data Per Outcome Measure at Baseline and 6 Months After Bariatric Surgery eTable 3. Covariates Included in Repeated Measures ANOVA Analysis eFigure 2. Changes in Cognitive Outcomes, Physical Activity and Depression Symptoms Among Patients Who Underwent Bariatric Surgery (n=133) eTable 4. Change in Plasma Markers Among Patients Who Underwent Bariatric Surgery (n=133) eReference. [file jamanetwopen-e2355380-s001.pdf]

Supplemental Online Content

Custers E, Vreeken D, Kleemann R, et al. Long-term brain structure and cognition following bariatric surgery. *JAMA Netw Open*. 2024;7(2):e2355380.
doi:10.1001/jamanetworkopen.2023.55380

eFigure 1. Flowchart of the Study

eMethods. Analysis of Adipokines and Cytokines and MRI Acquisition

eTable 1. Cortical Thinning Based on 0.6% Annual Normal Brain Aging Effects

eTable 2. Missing Data Per Outcome Measure at Baseline and 6 Months After Bariatric Surgery

eTable 3. Covariates Included in Repeated Measures ANOVA Analysis

eFigure 2. Changes in Cognitive Outcomes, Physical Activity and Depression Symptoms Among Patients Who Underwent Bariatric Surgery(n=133)

eTable 4. Change in Plasma Markers Among Patients Who Underwent Bariatric Surgery (n=133)

eReference.

This supplemental material has been provided by the authors to give readers additional information about their work.

© 2024 Custers E et al. *JAMA Network Open.*

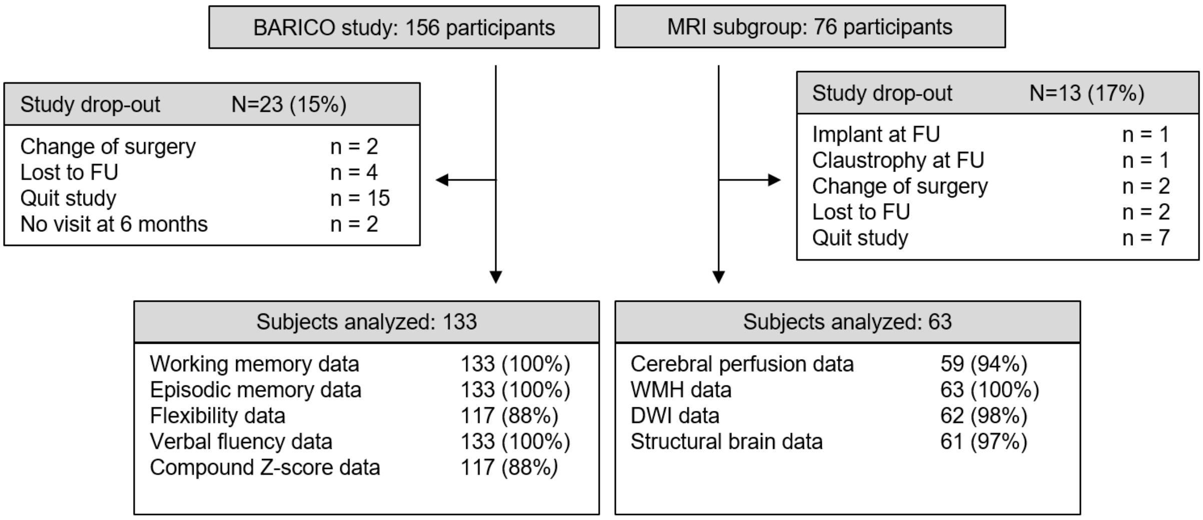

eFigure 1. Flowchart of the study. Due to technical issues of the Test of Attentional performance, data from 16 participants were missing for the flexibility data, which was consequently excluded from the compound Z-score data for these participants. Due to insufficient MRI quality, 4 participants were excluded for cerebral perfusion data, 1 participant for DWI data and 2 for structural brain data. Abbreviations: FU = follow-up, WMH = white matter hyperintensity, DWI = diffusion weighted imaging.

Analysis of adipokines and cytokines

Total leptin (pg/ml), adiponectin (µg/ml), high sensitive C-reactive protein (CRP, µg/ml)[1], serum amyloid A (SAA, µg/ml), plasminogen activator inhibitor-1 (PAI-1, ng/ml) and brain derived neurotrophic factor (BNDF, ng/ml) were determined with human enzyme-linked immunosorbent assays (ELISAs). Tumor necrosis factor alpha (TNF-α, pg/ml), interleukin-1β (IL-1β, pg/ml), IL-6 (pg/ml) and neurofilament light chain (NFL, pg/ml) were determined with single molecule array (SIMOA) technology using SP-X™ imaging system (Quanterix, Billerica, MA, USA).

MRI acquisition

Participants were scanned in a 3T Skyra scanner (Siemens Healthineers, Erlangen, Germany) using a 32-channel head coil. The protocol included a 3D T1-weighted magnetization-prepared rapid gradient-echo sequence (TR/TI/TE 2300/1100/3.03 ms; 8° flip angle; voxel size: 1 mm isotropic), a 3D fluid-attenuated inversion recovery (FLAIR) sequence (TR/TI/TE 5000/1800/397 ms; 120° flip angle; voxel size: 1 mm isotropic), a pulsed arterial spin labeling (ASL) sequence (PICORE, bolus duration: 700 ms; inversion time: 1800 ms; effective post-labeling delay: 1800-2140ms) with 2D echo-planar imaging readout (TR/TE 2500/12 ms; voxel size: 4×4mm, 9 slides of 8 mm with a 2 mm slice gap, 45 control-label pairs, no background suppression, mean control used as M0) and diffusion-weighted MRI scans using multiband echo-planar imaging (TR/TE 3275/91.4 ms; voxel size: 1.9 mm isotropic; 6× b=0 s/mm2, 42× b=900 s/mm2, 83× b=1800 s/mm2).

© 2024 Custers E et al. *JAMA Network Open.*

eTable 1. Cortical thinning based on 0.6% annual normal brain aging effects.

	Baseline	24 months	Normal aging 24 months (based on 0.6%)	Delta aging
Cingulate gyrus	2.398	2.369	2.374	0.005
Frontal cortex	2.552	2.510	2.527	0.017
Insula	2.884	2.859	2.855	-0.004
Occipital cortex	1.935	1.902	1.916	0.014
Parietal cortex	2.392	2.361	2.368	0.007
Temporal cortex	2.715	2.761	2.688	-0.073
GM	2.450	2.413	2.426	0.013

Normal aging at 24 months was calculated as followed: baseline value x 0.994 x 0.994. Brain regions with lower cortical thinning than the 0.6% normal age range (and thus less normal aging effects) are underscored.

© 2024 Custers E et al. *JAMA Network Open.*

eTable 2: Missing data per outcome measure at baseline and 6 months after bariatric surgery. N=133

Outcome measure	Number of missings (%)		
	Baseline	6 months	24 months
**WC[a]**	12 (9.0%)	22 (16.5%)	6 (4.5%)
**Blood pressure[a]**	0 (0%)	27 (20.3)	8 (6.0%)
**Plasma markers**			
hs-CRP	15 (11.3%)	3 (2.3%)	7 (5.3%)
Leptin	15 (11.3%)	3 (2.3%)	7 (5.3%)
Adiponectin	15 (11.3%)	3 (2.3%)	7 (5.3%)
SAA	15 (11.3%)	3 (2.3%)	7 (5.3%)
TNF-α	15 (11.3%)	3 (2.3%)	7 (5.3%)
IL-β	15 (11.3%)	3 (2.3%)	6 (4.5%)
IL-6	15 (11.3%)	3 (2.3%)	6 (4.5%)
PAI-1	15 (11.3%)	3 (2.3%)	6 (4.5%)
BDNF	15 (11.3%)	3 (2.3%)	6 (4.5%)
NFL	15 (11.3%)	3 (2.3%)	6 (4.5%)
**TAP Flexibility index score**	6 (4.5%)	9 (6.8%)	2 (1.5%)
**BDI[b]**	3 (2.3%)	5 (3.8%)	5 (3.8%)
**Baecke[b]**	10 (7.5%)	21 (15.8%)	23 (17.3)
**Cerebral perfusion data[c]**	4 (6.3%)		4 (6.3%)
**DWI data[c]**	1 (1.6%)		1 (1.6%)
**Structural brain data[c]**	2 (3.2%)		2 (3.2%)

Only parameters with missing data are represented in the table; all other parameters were complete at each time point. [a] We encountered substantial missing data for WC and blood pressure during the COVID-19 pandemic, as these measurements were no longer part of standard care due to social distance. [b] Many patients did not fill in the complete questionnaire and therefore these data were not reliable and these scores were excluded. [c] Due to insufficient MRI quality cases had to be excluded. Abbreviations: WC = waist circumference, hs-CRP = high sensitive C-reactive protein, SAA = serum amyloid A, TNF-α = tumor necrosis factor alpha, IL-1β = interleukin-1β, IL-6 = interleukin-6, PAI-1 = plasminogen activator inhibitor 1, BDNF = brain derived neurotrophic factor, NFL = neurofilament light chain, TAP = Tests of Attentional Performance, BDI = Beck Depression Inventory, DWI = diffusion weighted imaging.

© 2024 Custers E et al. *JAMA Network Open.*

eTable 3. Covariates included in repeated measures ANOVA analysis.

Variable	Covariate(s) used in analyses	Scientific rationale
Weight	Sex	Sex can influence weight
WC	Sex	Sex can influence WC
BMI	Sex	Sex can influence BMI
TBWL	Sex	Sex can influence TBWL
ICV nucleus accumbens	Education	Education can influence ICV
ICV cingulate cortex	Age	Age can influence ICV
ICV frontal cortex	Sex, education, preoperative BMI	Sex, education and BMI can influence ICV
ICV parietal cortex	Sex	Sex can influence ICV
CBF caudate nucleus	Postoperative head motion	Head motion can influence the CBF measures
CBF putamen	Postoperative head motion	Head motion can influence the CBF measures
CBF temporal cortex	Preoperative head motion, preoperative hematocrit	Head motion and hematocrit can influence the CBF measures
CBF nucleus accumbens	Postoperative head motion	Head motion can influence CBF measures
sCoV caudate nucleus	Preoperative BMI	BMI can influence sCoV
sCoV frontal cortex	Preoperative head motion, preoperative hematocrit	Head motion and hematcrit can influence sCoV
sCoV occipital cortex	Preoperative hematocrit, postoperative head motion	Head motion and hematcrit can influence sCoV
sCoV parietal cortex	Preoperative head motion, Preoperative hematocrit	Head motion and hematcrit can influence sCoV
sCoV temporal cortex	Preoperative hematocrit	Hematocrit can influence sCoV
MSMD	Sex	Sex can influence MSMD

Variables, at baseline and 24 months after bariatric surgery, have been divided by head motion and/or hematocrit values to correct for these covariates. Sex, age and education have been included in the repeated measures ANOVA analyses as a covariate. Abbreviations: WC = waist circumference, BMI = body mass index, TBWL = total body weight loss, ICV = intracranial volume, CBF = cerebral blood flow, sCoV = spatial coefficient of variation, WMH = white matter hyperintensities, MSMD = mean skeletonized diffusivity.

© 2024 Custers E et al. *JAMA Network Open.*

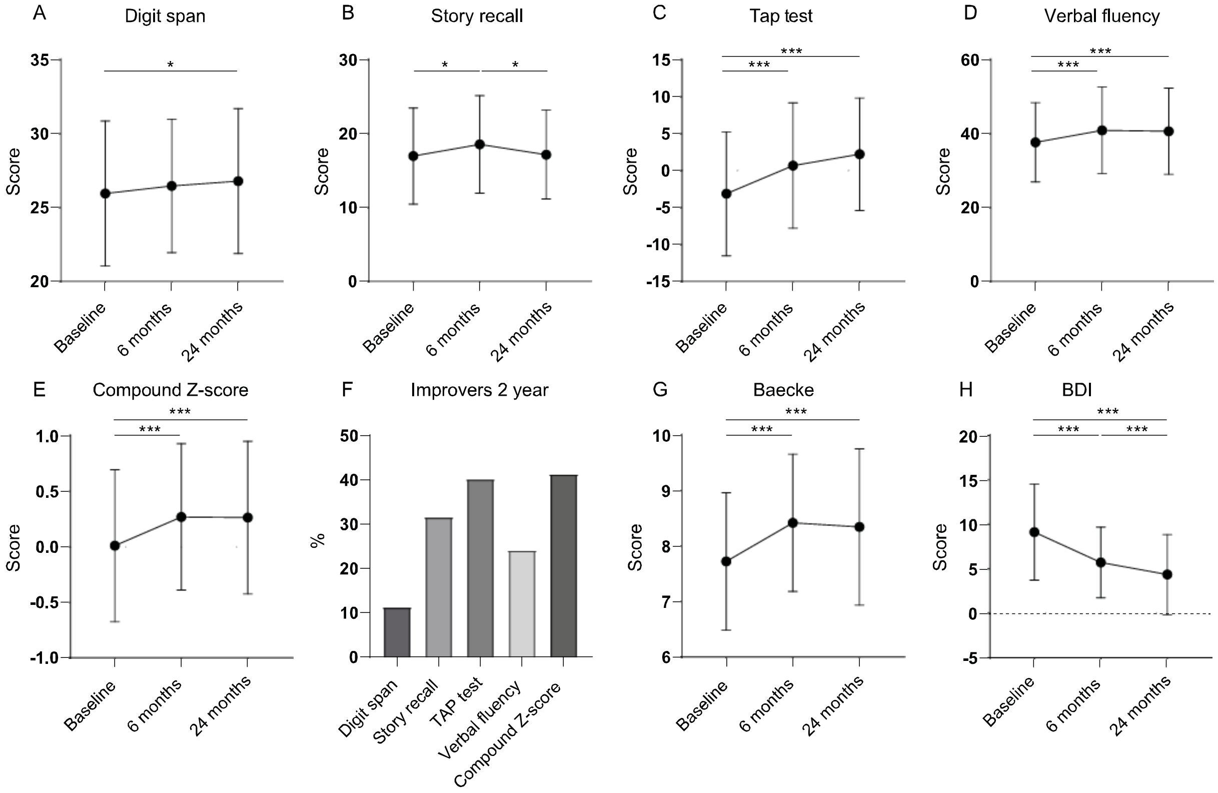

eFigure 2. Changes in cognitive outcomes, physical activity and depression symptoms among bariatric surgery patients (n=133). *Repeated measures analyses of variance were conducted to examine changes over time. Significant changes over time are indicated by stars. (a) Change in digit span (sum of forward, backward and sorting) over time. (b) change of story recall (sum of immediate and delayed recall) over time. (c) Change in TAP flexibility index score over time, complete data on all timepoints were available for 117 participants. (d) Change in controlled oral word association test over time. (e) Change in general cognition (based on compound Z-score) over time, complete data on all timepoints were available for 117 participants (f) Percentage of significant improvers for each cognitive domain separately and the compound Z-score based on the 20% index score. (g) Changes in physical activity over time, complete data on all timepoints were available for 91 participants. (h) Changes in depressive symptoms over time. Complete data on all timepoints were available for 124 participants. Values are presented as mean±SD, * p<0.05, ***p<0.001. Abbreviations: TAP = test of attentional performance, BDI = beck depression inventory.*

© 2024 Custers E et al. *JAMA Network Open.*

eTable 4: Change in plasma markers among bariatric surgery patients (n=133).

	Baseline	6 months	24 months	p-value
hs-CRP, median (IQR), ug/ml	4.77 (2.84-8.27)	1.47 (0.59-3.18)	0.80 (0.36-1.51)	<0.001
Leptin, median (IQR), pg/ml	65.75 (51.83-86.13)	14.05 (8.85-22.93)	14.25 (8.38-21.90)	<0.001
Adiponectin, median (IQR), ug/ml	2.30 (1.70-2.70)	2.40 (1.90-3.00)	3.45 (2.50-5.03)	<0.001
SAA, median (IQR), ug/ml	7.06 (5.09-17.11)	2.10 (1.21-4.29)	1.84 (1.35-3.08)	<0.001
TNF-α, median (IQR), pg/ml	3.89 (2.96-5.33)	3.57 (2.80-4.24)	3.11 (2.15-4.00)	<0.001
IL-1β, median (IQR), pg/ml	0.18 (0.06-0.34)	0.07 (0.01-0.20)	0.29 (0.17-0.46)	<0.001
IL-6, median (IQR), pg/ml	1.87 (1.37-3.02)	1.47 (1.08-2.20)	0.49 (0.28-1.06)	<0.001
PAI-1, median (IQR), ng/ml	40.62 (29.65-54.72)	29.82 (21.02-43.95)	43.49 (26.31-63.73)	<0.001
NFL, median (IQR), pg/ml	9.07 (7.36-11.14)	10.75 (8.90-13.87)	10.24 (8.33-13.26)	<0.001
BDNF, mean ± SD, ng/ml	17.15 ± 9.08	19.05 ± 8.77	23.55 ± 9.13	<0.001

Repeated measures analysis of variance were conducted to asses changes in brain parameters over time. Significant changes over time are indicated by underscoring the F and corresponding p-values Complete data for all parameters (except for IL-1β) on both timepoints were available for 110 participants. Complete data for IL-1β on both time points was available for 111 participants. Abbreviations: hs-CRP = high sensitive C-reactive protein, SAA = serum amyloid A, TNF-α = tumor necrosis factor α, IL = interleukin, PAI-1 = plasminogen activator inhibitor-1, NFL = neurofilament light chain, BDNF = brain derived neurotrophic factor, IQR = inter quartile range.

References

1. Schutte MH, Kleemann R, Nota NM, et al. The effect of transdermal gender-affirming hormone therapy on markers of inflammation and hemostasis. *Plos One*. 2022;17(3)doi:ARTN e026131210.1371/journal.pone.0261312

© 2024 Custers E et al. *JAMA Network Open.*
