# Supplementary material for: Long-Term Brain Structure and Cognition Following Bariatric Surgery
Source: JAMA Netw Open. 2024 Feb 9;7(2):e2355380. doi: 10.1001/jamanetworkopen.2023.55380 (PMC10858407; doi:10.1001/jamanetworkopen.2023.55380)
Supplement: Supplement 2. — Data Sharing Statement [file jamanetwopen-e2355380-s002.pdf]

Data Sharing Statement

Custers. Long-Term Brain Structure and Cognition Following Bariatric Surgery. *JAMA Netw Open*. Published February 09, 2024. doi:10.1001/jamanetworkopen.2023.55380

Data
Data available: No
